# Photodegradation of Buspirone: Ecotoxicological Assessment and Identification of Photoproducts

**DOI:** 10.3390/toxics14070633

**Published:** 2026-07-20

**Authors:** Grzegorz Nałęcz-Jawecki, Marta Kozak, Joanna Giebułtowicz, Milena Wawryniuk, Agata Drobniewska

**Affiliations:** 1 Department of Toxicology and Food Science, Medical University of Warsaw, Banacha 1 Str., 02-097 Warszawa, Polandmilena.wawryniuk@wum.edu.pl (M.W.); 2 Department of Drug Chemistry, Pharmaceutical and Biomedical Analysis, Medical University of Warsaw, Banacha 1 Str., 02-097 Warszawa, Poland; joanna.giebultowicz@wum.edu.pl

**Keywords:** pharmaceuticals in the environment, risk assessment, ecotoxicology

## Abstract

Buspirone is an anxiolytic drug used for the treatment of generalized anxiety disorder. Even though it was patented 50 years ago, it is still among the top three most commonly prescribed anti-anxiety medications in the US. Its fate in the environment and ecotoxic effects have not yet been assessed. In this study, the direct and indirect (with humic acids) photodegradation of buspirone was assessed using the SunTest CPS+ sunlight simulator in two radiation ranges: 280–800 nm and 290–800 nm. The concentration of buspirone and the formation of photoproducts were assessed using liquid chromatography with a diode array and mass spectrometry detector. Toxicity was assessed using biotests with luminescent bacteria and protozoan *Spirostomum ambiguum,* as well as in silico analyses on daphnia, fish, and protozoan *Tetrahymena pyriformis*. Buspirone was degraded by sunlight. Expanding the radiation range from 290 to 280 nm accelerated degradation. Humic acids, however, had no effect in either case. The structures of 24 phototransformation products have been proposed. Neither buspirone nor most of its photodegradation products were toxic. Only two derivatives had a calculated 96h-LC_50_ value for fish below 1 mg L^−1^. The obtained results indicate a low environmental risk of buspirone.

## 1. Introduction

Buspirone (8-{4-[4-(Pyrimidin-2-yl)piperazin-1-yl]butyl}-8-azaspiro[4.5]decane-7,9-dione, ATC code N05BE01, BSP) is an anxiolytic drug used to treat generalized anxiety disorder. Even though it was patented 50 years ago, it is still among the three most commonly prescribed anti-anxiety medications in the US. Its use has steadily increased, as it causes fewer side effects than benzodiazepines and does not induce addiction. Its position on the list of most frequently sold drugs in the US rose from 106th to 40th place between 2015 and 2023, and the annual number of prescriptions exceeded 15 million [1]. BSP is used in veterinary medicine to treat pets and has been illegally administered to calm high-spirited horses during the race [2,3]. The BSP’s mechanism of action involves interaction with the serotonin 5-HT1A receptor and interaction with a dopamine D2 receptor [4]. Thus, it can affect all organisms that possess these receptors including invertebrates and fish. BSP influenced fish behavior, attracted the zebrafish (*Danio rerio*) at 1 mg L^−1^ [4], and increased zebrafish exploration of higher portions of the aquaria [5]. However, unlike widely studied serotonin selective reuptake inhibitors (SSRIs), both the effects of BSP on aquatic organisms and its fate in the environment are mainly unknown.

Photodegradation is one of the major abiotic processes influencing the fate of organic contaminants in surface water. In the process of direct photodegradation, light acts directly on molecules that absorb radiation, leading to their transformation. In indirect photodegradation, photons are absorbed by natural organic matter (NOM) dissolved in water, e.g., humic acids (HA) [6]. The reactive intermediates formed in this process, such as reactive oxygen species, may induce chemical transformations of xenobiotics. The process of photodegradation under the influence of solar radiation rarely leads to complete degradation of the substance. Most often, various photodegradation products are formed, of unknown toxicity, even higher than that of the parent compound [7,8]. Therefore, when assessing photodegradation processes, in addition to chemical methods enabling the identification of photoproducts, it is necessary to use biological methods, i.e., biotests [9,10,11].

**Figure 1:**
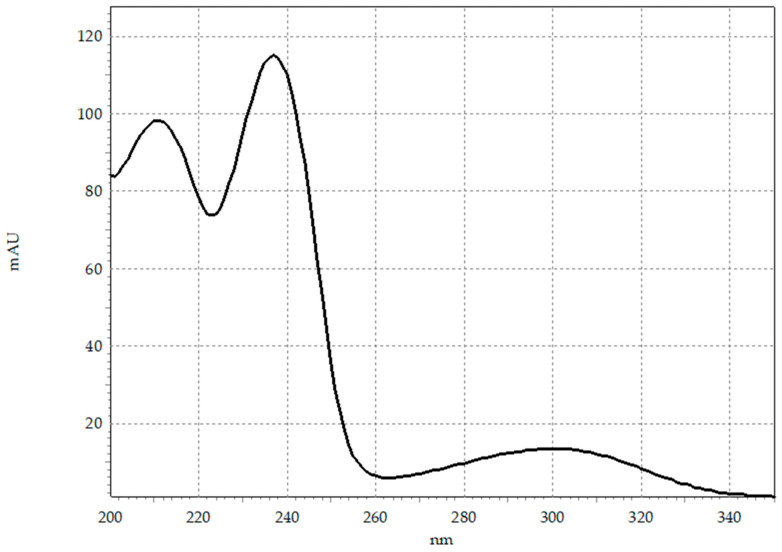
UV-Vis spectrum of BSP.

Sunlight with wavelengths above 280–290 nm reaches the Earth’s surface. The absorption spectrum of BSP (Figure 1) indicates that this compound absorbs UV light up to 340 nm and is therefore susceptible to direct photodegradation. Khedr and Sakr [12] analyzed BSP stability in pharmaceutical formulations and found that it undergoes slow degradation under sunlight exposure. Ruokolainen et al. [13] and Calza et al. [2] used titanium dioxide as a photocatalyst to mimic BSP metabolic reactions, and identified 7 and 13 degradation products, respectively. However, the photodecomposition of BSP in the aquatic environment under the influence of sunlight has not been assessed so far.

The aim of this study is to fill the existing knowledge gap about the fate of BSP in the environment and its effects on aquatic organisms. In this work, direct and indirect photodegradation of BSP was evaluated using the SunTest CPS+ light simulator. The concentration of BSP during irradiation was determined by HPLC with a diode array detector. Photodegradation products were identified by mass spectrometry. High concentrations of BSP were used in this study to obtain accurate chemical analysis data without the need for sample preparation. Concentrating solutions, for example, through extraction, could change their composition, especially if the derivatives studied differed in polarity.

The ecotoxicity of BSP solutions before and after degradation was assessed using bioassays with the luminescent bacterium *Aliivibrio fischeri* and protozoa *Spirostomum ambiguum*. Additionally, the ecotoxicity of the resulting photoproducts was estimated in silico using the Toxicity Estimation Software Tool from U.S. EPA.

## 2. Materials and Methods

### 2.1. Chemicals

The BSP hydrochloride was purchased from Merck (Darmstadt, Germany). The chemical was of high purity and was stored at −20 °C in the original glass bottle. The BSP working solution (40 mg L^−1^) was prepared immediately before the experiments by diluting the standard solution with a suitable diluent. Tyrode’s medium was used as a diluent and as the negative control. It is a substitute for soft water with low mineral content and it consists of: 125 mg NaCl, 3.1 mg KCl, 3.1 mg CaCl_2_, 1.55 mg MgCl_2_, 15.6 mg NaHCO_3_, and 0.78 mg NaH_2_PO_4_ per liter of deionized water. The pH of Tyrode’s medium was 7.4 ± 0.2 [14]. A stock solution of humic acids from Merck (Darmstadt, Germany) (HA) was prepared according to the U.S. EPA guideline [15]. The HA stock solution was diluted 1000-fold just before the test. The absorbance of the working solution measured in the Shimadzu 1601 spectrophotometer (Kyoto, Japan) was 0.05 at 370 nm. Hydrogen peroxide (30%) was from POCH (Gliwice, Poland). To obtain an H_2_O_2_ concentration of 1 µM, 10 µL of stock solution was added to 10 mL of sample just before the test.

### 2.2. Photodegradation Experiment

The irradiation of samples was carried out using the SunTest CPS+ apparatus (Klimatest, Warsaw, Poland) equipped with a 1500 W xenon lamp. Two experimental variants were applied: without a filter—radiation in the range of 280–800 nm (UV-1) and with the UV filter (UV-2), which limits the ultraviolet range to the range of solar radiation reaching the Earth’s surface (290–800 nm). The fluence rate was set to 750 W m^−2^, which corresponds to a dose of 2700 kJ m^−2^ h^−1^. For the photodegradation experiments, BSP working solutions (40 mg L^−1^) in Tyrode’s medium with and without HA were irradiated in 60 mL quartz tubes in a temperature-controlled chamber (30–35 °C) for 4 h. The subsamples for chromatographic analyses were collected every 1 h during irradiation. As a dark control, the same solutions were kept in the dark for 4 h. The test was repeated three times.

### 2.3. Liquid Chromatography

The concentration of BSP was determined using a HPLC chromatograph with an SPD-M10A diode-array detector (HPLC-DAD) from Shimadzu (Kyoto, Japan). In the applied gradient method, mobile phase A consisted of a 0.05% aqueous solution of trifluoroacetic acid (Merck, Darmstadt, Germany), whereas mobile phase B consisted of HPLC-gradient grade acetonitrile (Merck, Darmstadt, Germany).

Deionized water was from a Milli-Q^®^ Direct water purification system (Merck, Darmstadt, Germany). Separation was performed on a LichroCART 55 × 4 Purospher STAR RP-18 (3 µm) analytical column (Merck, Darmstadt, Germany). The flow rate was 1.0 mL min^−1^, and the concentration of phase B during the analysis changed according to the following scheme: 0 min: 25%; 1 min: 40%; 5 min: 90%; 6 min: 90%; and 6.10 min: 25%. Quantitative analysis of BSP was carried out at 237 nm. A standard curve of BSP solutions was constructed in the concentration range of 0.05–10 mg L^−1^. The limit of quantitation was 0.05 mg L^−1^.

Photoproducts formed during sample irradiation were searched using an untargeted method with UHPLC Dionex Ultimate 3000 with a Q-Exactive hybrid quadrupole-orbitrap mass spectrometer system from Thermo Fisher Scientific, Waltham, MA, USA (MS/MS), as described previously [10]. Tentative metabolites were detected using Compound Discoverer Software, v.2.1 (Thermo Fisher Scientific, Waltham, MA, USA).

### 2.4. Toxicity Assays

The acute toxicity of the samples was evaluated using the Spirotox and luminescent bacteria assays. The Spirotox test with the ciliate protozoan *S. ambiguum* was performed according to the standard operational procedure [14]. Briefly, the tests were performed in polystyrene 24-well plates, with two replicates per plate. A series of five 2-fold dilutions of the test samples and a negative control were prepared directly in the multiwells. Ten organisms were introduced into each well containing 1 mL of the tested sample. After 24 h incubation at 25 °C in the dark, test effects, including morphological deformities and mortality, were observed with a dissection microscope. The luminescent bacteria assay was carried out according to the ISO 11348-3 [16] guideline with freeze-dried *A. fischeri* bacteria purchased from Modern Water (Sand Hutton, York, UK). The toxicity of two types of samples was assessed: non-irradiated and irradiated.

Toxicity Estimation Software Tool from U.S. EPA (T.E.S.T., v.5.1.2, Washington DC, USA) was used to predict the ecotoxicity of BSP transformation products formed during its irradiation. Fathead minnow fish (*Pimephales promelas*) 96h-LC_50_, *Daphnia magna* 48h-LC_50,_ and *Tetrahymena pyriformis* 48h-IGC_50_ endpoints data were calculated with the consensus method.

## 3. Results

### 3.1. The Concentration of BSP During Irradiation

**Figure 2:**
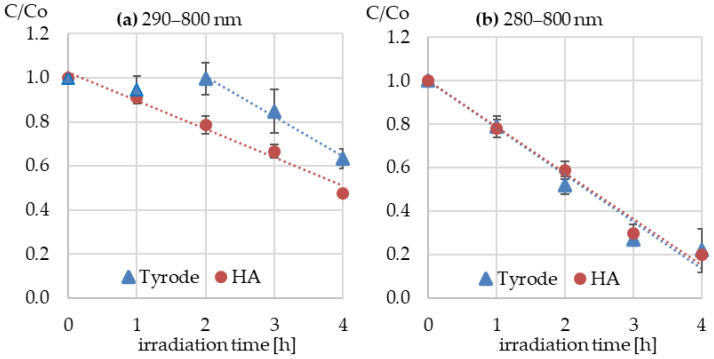
Concentration of BSP irradiated in Tyrode’s medium without (Tyrode) and with humic acids (HA) in SunTest CPS+ (**a**) with UV filter (290–800 nm) and (**b**) without any filter (280–800 nm).

BSP solutions in Tyrode’s medium with and without HA were exposed in the SunTest CPS+ apparatus to two radiation ranges: UV-1 (280–800 nm) and UV-2 (290–800 nm). The concentration of the BSP was determined with the HPLC-DAD. No loss of the BSP was observed in the dark controls. During the first 2 h of UV-1 irradiation, the BSP concentration remained unchanged (Figure 2a). However, during the subsequent irradiation period, it gradually decreased to 63% of the initial value. However, in the presence of HA, BSP gradually decomposed, and after 4 h, the concentration decreased to 47% of the initial value. The BSP degradation reaction followed pseudo-first-order kinetics, and the rate constant values were −0.182 h^−1^ (R^2^ = 0.988) and −0.130 h^−1^ (R^2^ = 0.981), for solutions without (time period 2–4 h) and with humic acids, respectively.

Expanding the radiation range from 290 to 800 nm to 280–800 nm significantly accelerated the decomposition of the drug, and after 4 h, the BSP concentration decreased to 20% of the initial value, both in the presence and absence of HA (Figure 2b). In both cases, the degradation reaction also followed pseudo-first-order kinetics, and the reaction rate constant values were practically the same and were −0.216 h^−1^ (R^2^ = 0.966) and −0.211 h^−1^ (R^2^ = 0.985), in the presence and absence of HA, respectively.

The calculated half-life of BSP was 2.3 h and 4.1 h for UV-1 and UV-2 irradiation, respectively. This means that even a small shift in the radiation range towards shorter wavelengths can significantly accelerate the decomposition of the substance.

### 3.2. Photoproducts Formed During Irradiation

**Figure 3:**
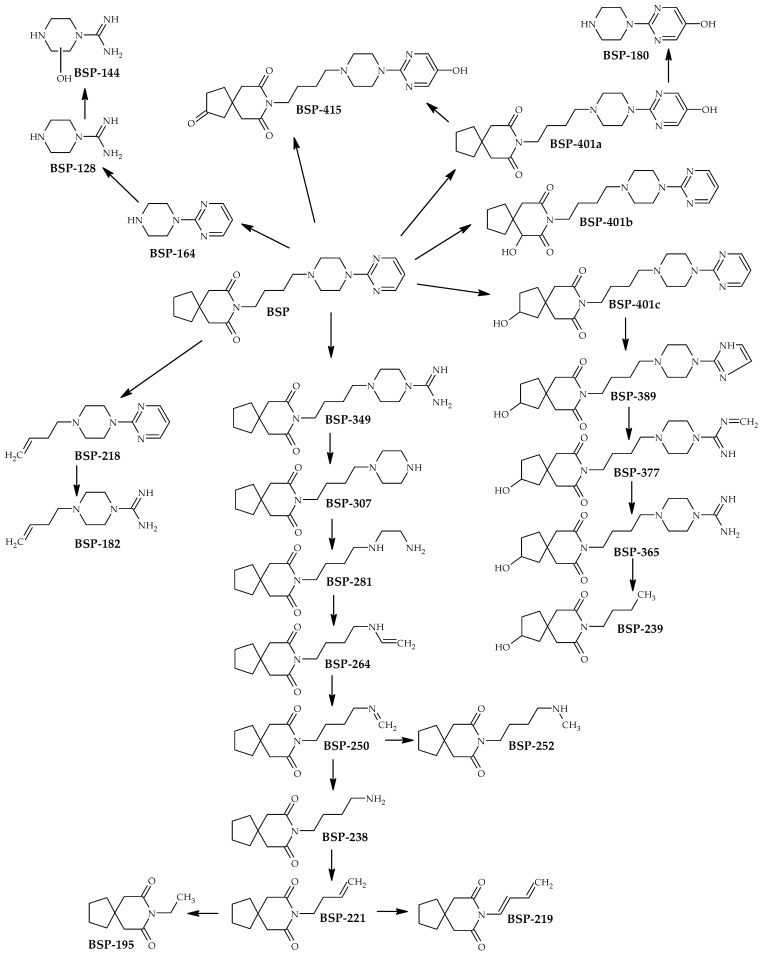
Proposed photodegradation pathway of buspirone.

Among the compounds detected by LC-MS/MS, 24 showed abundances that were more than 100-fold higher in the irradiated samples than in the initial BSP samples. Based on molecular masses and MS2 fragmentation, the structures of these products and probable transformation pathways were proposed (Table 1, Figure 3).

Three structures with masses of 401.2427 were detected and attributed to hydroxy buspirone (BSP-401). MS2 fragmentation indicates that they differ in the site of hydroxyl substitution (Figure 3). Both BSP and hydroxy buspirone underwent gradual degradation, first of the pyrimidine ring and then of the piperazine ring. The analysis also revealed the presence of 1-pyrimidinyl piperazine (BSP-164), its hydroxy- (BSP-180) and N-alkyl derivatives (BSP-218 and BSP-182), and its degradation products (BSP-128 and BSP-144). Additionally, we observed oxo hydroxy buspirone (BSP-415).

All these derivatives were observed both in samples irradiated without and with the addition of HA, as well as in samples irradiated with a narrower (290–800 nm) and a wider (280–800 nm) range of UV-Vis radiation. This means that the change in the radiation range and the presence of HA only influenced the intensity of the observed peaks.

### 3.3. The Effect of Irradiation on the BSP Toxicity

**Figure 4:**
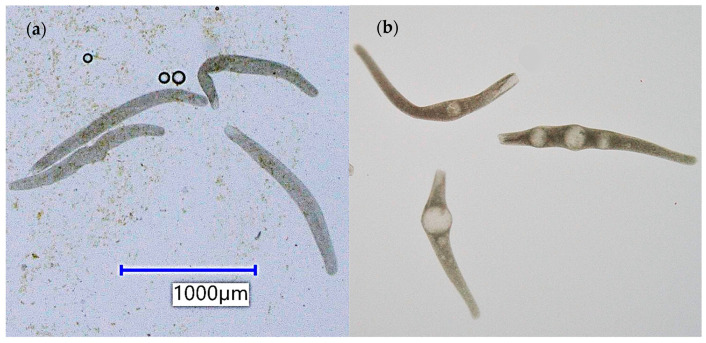
Protozoan *S. ambiguum* (**a**) normal cells; (**b**) treated with BSP 20 mg L^−1^. Photograph taken with the Keyence VHX 7000 digital microscope.

The acute toxicity of BSP toward luminescent bacteria and protozoa was low. Tested drug concentrations up to 40 mg L^−1^ caused neither a decrease in bacterial luminescence nor mortality in the protozoan species. However, at the two highest BSP concentrations, 20 and 40 mg L^−1^, the formation of large vacuoles in *S. ambiguum* cells was observed (Figure 4). This test effect in protozoa has not been observed in previous studies. Toxicity towards luminescent bacteria and protozoa did not change during irradiation of BSP solutions, both in Tyrode’s medium and in the presence of HA.

**Figure 5:**
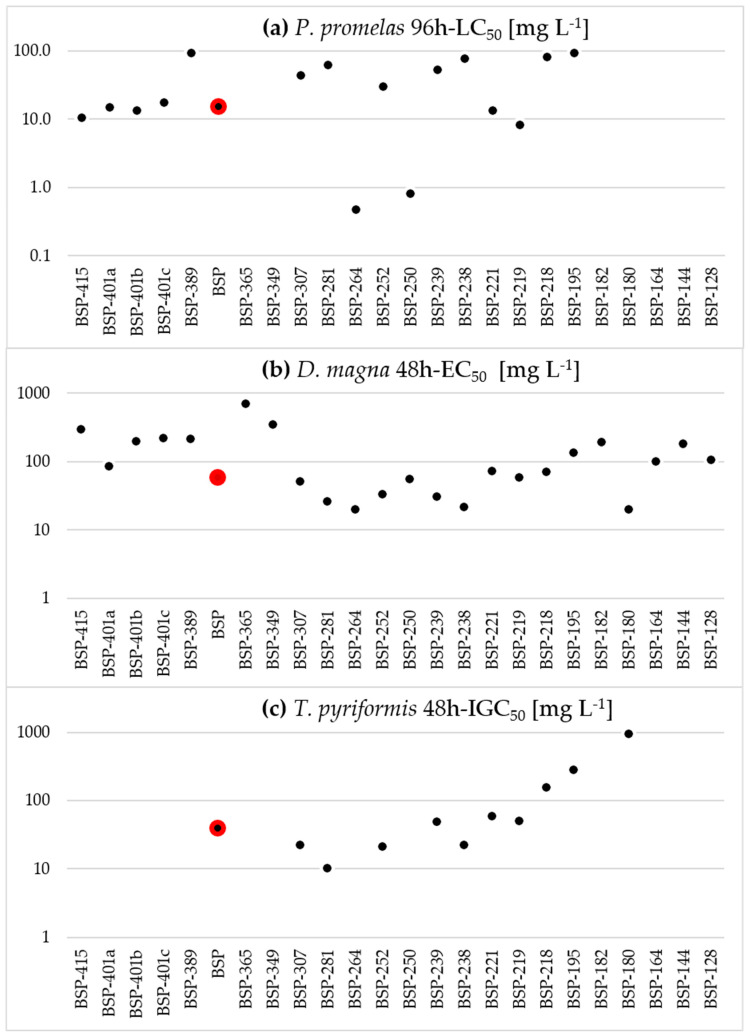
Toxicity of BSP and its photoproducts generated in the T.E.S.T. software (v.5.1.2). (**a**) *P. promelas* 96h-LC_50_; (**b**) *D. magna* 48h-EC_50_; (**c**) *T. pyriformis* 48h-IGC_50_.

The toxicity of BSP predicted using the T.E.S.T. program was also low for all three tested organisms. The median toxicity values ranged from 15.3 mg L−1 for fathead minnow fish, to 40.0 mg L−1 for the protozoan *T. pyriformis*, and 58.3 mg L−1 for the crustacean *D. magna* (Figure 5). The predicted toxicity of most of the phototransformation products was in a similar range to that of BSP. However, for two derivatives, BSP-264 and BSP-250, the predicted 96h-LC_50_ value for fish was very low and amounted to 0.48 mg L^−1^ and 0.83 mg L^−1^, respectively.

## 4. Discussion

### 4.1. Toxicity of BSP

The environmental risk assessment of pharmaceuticals comprises two stages: an exposure analysis and an analysis of environmental fate and effects on organisms [17]. Despite its widespread use, BSP has not yet been detected in the aquatic environment, primarily because it was not among the pharmaceuticals monitored in wastewater and surface water [18,19,20]. Its toxic effects have not yet been assessed in standard biotests recommended by the EMA guideline [17]. Targeted studies have been conducted on fish to assess the effects associated with their biological mechanism of action, namely interactions with serotonin receptors. Behavioral changes were observed at concentrations of 1 mg L^−1^ [4,21,22]. In this work, two biotests on unicellular organisms, widely used in ecotoxicology, were used. Bacteria and protozoa play a key role in water and wastewater treatment processes. Spirotox bioassay with protozoa is highly sensitive to a number of toxic substances, including drugs affecting the nervous system, i.e., antipsychotics [23] and antidepressants [24]. Luminescent bacteria, on the other hand, are the most commonly used biotest, and their sensitivity, related to basic, general, non-specific biological activity, is similar to that of higher organisms [25]. We used high concentrations of BSP for testing to capture potential toxic effects. The results obtained in this study indicate low toxicity of BSP toward both protozoa and luminescent bacteria. NOEC values are significantly higher than those for antidepressants and antipsychotics, for which they are below 1 mg L^−1^ [23,24]. The low acute toxicity of BSP is confirmed by in silico calculations using T.E.S.T. software, in which the median toxicity values ranged from 15.3 mg L^−1^, through 40.0 mg L^−1^ to 58.3 mg L^−1^ for *P. promelas*, *T. pyriformis*, and *D. magna*, respectively.

The analysis of the environmental risk of xenobiotics, including active pharmaceutical ingredients (API), requires a comparison of the predicted (PEC) or measured (MEC) concentration of the substance in the environment with the predicted no-toxicity concentration (PNEC). The PNEC should be calculated based on the results of chronic tests on organisms from three trophic levels, although in their absence, it is possible to use an assessment factor (AF) [17]. Considering the AF of 1000, the PNEC value for BSP is around 10 µg L^−1^. Unfortunately, without data on BSP concentrations in surface waters, it is impossible to calculate the environmental risk value. Considering the fact that API concentrations in rivers rarely exceed 1 µg L^−1^, this risk can be considered small. However, a proper risk assessment requires knowledge of the missing data [17].

### 4.2. Phototransformation of BSP

Photodegradation of pharmaceutically active compounds (PhACs) is a major abiotic degradation process occurring in sunlit freshwater environments. Understanding of this process is important for characterizing the fate of PhACs in water, and their effects on non-target organisms [26]. Photodegradation of only some PhACs is well-studied [8,26]. For many pharmaceuticals, even those used in large quantities, data on photolability in an aqueous environment and the ecotoxicity of photoproducts are lacking. The present study aims to fill the knowledge gap on BSP photodegradation. In this study, we used high concentrations of BSP to analyze solutions without prior concentration, which could result in the loss of compounds of different polarity. Due to the lack of data on actual BSP concentrations in the aquatic environment, lower concentration levels were not used. It is important to be aware that photodegradation of the substance in natural waters may proceed differently due to lower concentrations and the coexistence of other substances that may affect this process.

After 4 h of BSP irradiation with light in the range of 290–800 nm, the drug concentration decreased by 37%, and a lag phase was observed during the first 2 h of exposure. The presence of HA resulted in the absence of this lag phase, but the degradation of BSP after 4 h was only 16% greater. It is unclear what caused the lag phase. The observed lag phase can be explained in two ways. First, the BSP degradation reaction may require high activation energy, i.e., a higher concentration of reactive species. Second, by the formation of a BSP photodegradation product with a retention time and UV spectrum very similar to BSP, which would falsify the quantification of BSP using the LC technique with the diode array detector. Distinguishing whether we were dealing with a real phase delay or a separation error of the HPLC analysis with a diode array detector may have required the use of more advanced techniques, which unfortunately were not available, e.g., a different detector (triple quadrupole MS). Chen et al. [27], while studying photoassisted Fenton oxidations of aromatic compounds, observed a lag phase, which they explained by the formation of an appropriate concentration of reactive intermediates.

Humic acids are naturally occurring colored substances that can act in two ways by absorbing radiation [28,29,30]. On the one hand, they can act as photosensitizers. Through the generation of reactive oxygen species, they can cause the phototransformation reactions of other compounds [28,29,30]. They can then cause the decomposition of substances that do not absorb radiation in a given range (indirect photodegradation). On the other hand, by absorbing radiation in the same spectral range and/or scavenging reactive species, they can act as inner filters, slowing down the direct photodegradation reaction [29,30]. When exposed to light in the 280–800 nm range, the presence of HA did not affect the transformation of BSP. This indicates that direct photodegradation occurred, caused by direct absorption of radiation by the BSP. However, in the narrower radiation range of 290–800 nm, the presence of HA caused the reaction to start more quickly, perhaps by lowering the activation energy.

As a result of irradiation, with decreasing BSP concentration, the formation of several transformation products was observed in both HPLC-DAD and LC-MS/MS chromatograms. Based on HR LC-MS/MS results, accurate masses, isotopic composition, and fragmentation spectra, plausible structures of 24 photoproducts, and probable transformation pathways were proposed. Due to the lack of analytical standards and the high complexity of the reaction mixture, the proposed structures could not be confirmed. The degradation of BSP has been studied in various systems [2,13,31]. Ruokolainen et al. [13], while studying the photocatalytic transformations of BSP under the influence of TiO_2_ and UVA (365 nm), detected seven derivatives. Zhang et al. [26], studying the metabolism of BSP in rat microsomes, identified 11 metabolites, while Stanley et al. [3] analyzed the metabolism of BSP in horses, and they found the presence of at least three metabolites. The compounds formed during photodegradation and photocatalysis are in many cases identical to metabolites in mammals [2]. As a result of irradiation, three hydroxyl derivatives of BSP were formed, differing in the place of substitution of the -OH group. This is consistent with data obtained from photocatalysis [2,13]. BSP-415, probably oxo-hydroxy-buspirone, is the only BSP derivative with a higher number of oxygen atoms. Dihydroxy-buspirone and oxy-hydroxy-buspirone were detected as minor products during TiO_2_-assisted photocatalysis of BSP [13]. Calza et al. [2] also identified both BSP oxo isomers and their two dihydroxy derivatives. Photodegradation of the BSP and hydroxy-buspirone molecules proceeds primarily through the gradual degradation of the pyrimidine ring and then the piperazine ring. The detection of intermediate degradation products allowed us to propose a transformation pathway (Figure 3). The presence of the *m*/*z* 222.149 molecular ion evidences the intact butyl-azaspirone-decanedione structure. The proposed transformation pathway is consistent with previous studies that showed the presence of despyrimidinyl-hydroxy-buspirone-amidine (BSP-365), despyrimidinyl-buspirone-amidine (BSP-349), despyrimidinyl-buspirone (BSP-307) [2], and BSP-264 [31]. Cleavage of the BSP molecule leads to the formation of BSP-218 and BSP-164. While 1-pyrimidinyl piperazine (BSP-164) had been reported previously as one of the BSP transformation products [2], the plausible structure of BSP-218 is proposed for the first time.

### 4.3. Toxicity of BSP Phototransformation Products

The toxicity of PhAC transformation products cannot be determined individually, as these compounds occur in the post-reaction mixture and are rarely commercially available. Therefore, the toxicity of the irradiated drug solutions was often compared to the toxicity of the unirradiated solution using tests measuring non-specific toxicity, e.g., luminescent bacteria [32]. In our studies, the toxicity of BSP solutions, both before and after irradiation, was low. In the case of luminescent bacteria, no reduction in luminescence was observed even at concentrations of 40 mg L^−1^. However, for the protozoan *S. ambiguum*, we observed the development of large vacuoles in BSP solutions at concentrations of 40 and 20 mg L^−1^. Similar effects were also observed in irradiated solutions, which may suggest that the products of phototransformation may be slightly toxic.

In silico analysis of BSP phototransformation products showed that most of them exhibited toxicity comparable to that of the parent compound. Only two products, BSP-264 and BSP-250, were predicted to be toxic to fish with 96h-LC_50_ < 1 mg L^−1^. Baran et al. [33], studying the in silico toxicity of enrofloxacin photoproducts, also observed high toxicity to fish of two photoproducts, despite the very low toxicity of the parent compound. In our previous studies [9], thanks to the possibility of purchasing the duloxetine toxic photoproduct identified by in silico analysis, we managed to confirm its high biological activity towards protozoa. Unfortunately, neither BSP-264 nor BSP-250 is commercially available.

In silico analyses predict a compound’s toxicity based on its structure, underlying physicochemical properties, and existing toxicological data of similar compounds. However, as the structure becomes more complex, specific features may emerge. Especially in the case of pharmacologically active compounds, specific interactions with receptors present in cells may occur. Therefore, the results of in silico analyses should be treated with great caution and, whenever possible, confirmed using tests on living organisms.

## 5. Conclusions

The first environmental studies of BSP, a widely used anxiolytic drug, have shown that it is degraded by UV-Visible light in the 290–800 nm range. A slight extension of the radiation range (280–800 nm) significantly accelerated BSP degradation. However, the addition of humic acids does not affect the drug’s degradation. Irradiation of BSP causes hydroxylation of the molecule and also gradual degradation, first of the pyrimidine ring and then of the piperazine ring. Acute toxicity analysis on luminescent bacteria and protozoa, as well as in silico, showed low toxicity of BSP and most of its phototransformation products. Therefore, it can be assumed that BSP does not pose an acute environmental risk. However, to accurately assess the environmental risk of BSP, it is necessary to conduct chronic toxicity studies and determine the actual concentrations of BSP in the environment.

In the present study, the formation of large vacuoles was observed in the protozoa *S. ambiguum*. In further studies, it would be interesting to understand the mechanism of this biological response and to determine whether it also occurs in other cells (including mammalian cells) following exposure to BSP.

## Figures and Tables

**Table 1:** Tentative identification of BSP and its degradation products. Identification was performed based on *m*/*z*, isotopic pattern, and fragmentation spectra (coverage by in silico fragmentation).

NAME	FORMULA	CALC. MW ^1^	Δ MASS [PPM]	RDBE ^2^	H/C ^3^	SELECTED MS/MS FRAGMENTS [*m*/*z*]
**BSP-415**	C_21_H_29_N_5_O_4_	415.2220	0.14	10.0	1.4	398.2194; 370.2242; 152.1072; 109.0650
**BSP-401A**	C_21_H_31_N_5_O_3_	401.2427	0.19	9.0	1.5	374.2550; 293.1860; 136.0870
**BSP-401B**	C_21_H_31_N_5_O_3_	401.2427	0.08	9.0	1.5	384.2395; 222.1491; 164.0820; 122.07133
**BSP-401C**	C_21_H_31_N_5_O_3_	401.2427	0.50	9.0	1.5	384.2404; 277.1910; 222.1488; 122.0713
**BSP-389**	C_20_H_31_N_5_O_3_	389.2427	0.34	8.0	1.6	374.2193; 222.1490; 152.1071; 98.0713
**BSP**	**C_21_H_31_N_5_O_2_**	**385.2478**	0.24	**9.0**	**1.5**	**308.2331; 222.1488; 152.1072; 86.0713**
**BSP-377**	C_19_H_31_N_5_O_3_	377.2427	0.82	7.0	1.6	308.2331; 222.1488; 152.1070; 97.0744
**BSP-365**	C_18_H_31_N_5_O_3_	365.2427	0.06	6.0	1.7	306.2176; 251.1755; 222.1492; 112.0870
**BSP-349**	C_18_H_31_N_5_O_2_	349.2478	0.50	6.0	1.7	308.2332; 222.1489; 112.0870; 86.0713
**BSP-307**	C_17_H_29_N_3_O_2_	307.2260	0.72	5.0	1.7	265.1908; 222.1488; 152.1070; 109.0648
**BSP-281**	C_15_H_27_N_3_O_2_	281.2103	0.48	4.0	1.8	265.1912; 222.1491; 152.1072; 98.0965
**BSP-264**	C_15_H_24_N_2_O_2_	264.1838	0.19	5.0	1.6	222.1491; 152.1072; 95.0855; 81.0700
**BSP-252**	C_14_H_24_N_2_O_2_	252.1838	0.63	4.0	1.7	No spectrum
**BSP-250**	C_14_H_22_N_2_O_2_	250.1681	0.15	5.0	1.6	222.1490; 152.1073; 95.0856; 84.0809
**BSP-239**	C_13_H_21_NO_3_	239.1521	0.31	4.0	1.6	222.1488; 153.0912; 109.0649; 81.0699
**BSP-238**	C_13_H_22_N_2_O_2_	238.1681	0.55	4.0	1.7	222.1491; 152.1072; 95.0855; 72.0809
**BSP-221**	C_13_H_19_NO_2_	221.1416	0.90	5.0	1.5	180.1019; 152.1071; 109.0648; 81.0699
**BSP-219**	C_13_H_17_NO_2_	219.1259	0.43	6.0	1.3	176.1073; 132.0811; 112.0759; 95.0492
**BSP-218**	C_12_H_18_N_4_	218.1531	0.05	6.0	1.5	177.1132; 148.0868; 122.0711; 98.0963
**BSP-195**	C_11_H_17_NO_2_	195.1259	0.53	4.0	1.5	145.1086; 128.0818; 103.0866; 86.0838
**BSP-182**	C_9_H_18_N_4_	182.1531	0.02	3.0	2.0	166.1339; 141.1386; 124.1121; 99.0916
**BSP-180**	C_8_H_12_N_4_O	180.1011	0.25	5.0	1.5	163.0984; 137.0603; 70.0655; 58.0656
**BSP-164**	C_8_H_12_N_4_	164.1062	0.60	5.0	1.5	149.0827; 122.0715; 96.0558; 81.04474
**BSP-144**	C_5_H_12_N_4_O	144.1011	<0.01	2.0	2.4	128.0818; 103.0866; 85.0762; 60.0560
**BSP-128**	C_5_H_12_N_4_	128.1062	0.82	2.0	2.4	112.0870; 85.0761; 87.0918; 70.0652

^1^ Monoisotopic molecular mass; ^2^ Rings and double bonds equivalent; ^3^ Hydrogen versus carbon atoms ratio.

## Data Availability

The data supporting the reported results are stored at the Department of Toxicology and Food Science, Medical University of Warsaw, and are not publicly available. The data are available from the corresponding authors upon reasonable request.
